# Statistical optimisation and analysis of biomass and exopolysaccharide production by *Lacticaseibacillus rhamnosus* LRH30

**DOI:** 10.1007/s11274-025-04273-2

**Published:** 2025-01-31

**Authors:** Helena Mylise Copeland, Susan Maye, George MacLeod, Dermot Brabazon, Christine Loscher, Brian Freeland

**Affiliations:** 1 School of Biotechnology, Dublin City University, Dublin, D9 Ireland; 2 I-Form, Advanced Manufacturing Research Centre, Dublin City University, Dublin, D9 Ireland; 3 Dairygold Co-Operative Society Limited, Clonmel Road, Co. Cork, Mitchelstown, P67 DD36 Ireland

**Keywords:** Design of experiments, Bioprocess, Functional foods, *Lacticaseibacillus rhamnosus*, Exopolysaccharide, Immunomodulatory

## Abstract

Exopolysaccharides (EPS) produced by lactic acid bacteria with immunomodulatory potential are promising natural food additives. This study employs small-scale, 250 mL bioreactors combined with a central composite design to optimise two important bioprocess parameters, namely temperature and airflow, to achieve high yields of biomass and EPS from *Lacticaseibacillus rhamnosus* LRH30 (*L. rhamnosus* LRH30). A quadratic model was determined to be the best fit for the production of both products. The optimum critical process parameters for maximised biomass were identified to be 37.01 °C with an airflow of 0.12 vvm, while optimum criteria was 20.1 °C with an airflow of 0.18 vvm for maximum EPS production. Under these optimized conditions, small-scale batch experiments yielded a biomass concentration of 10.1 g/L and an EPS yield of 520.2 mg/L. In comparison, scale-up experiments in 2L reactors resulted in a biomass concentration of 8.54 g/L (a reduction of 18%) and an EPS yield of 654.6 mg/L (an increase of 26%). The produced EPS was purified and characterised through Fourier transform infrared spectroscopy and showed characteristic peaks associated with polysaccharides. The immunomodulatory potential of the *L. rhamnosus* LRH30 cells and EPS was evaluated through cytokine and chemokine secretion in a J774A.1 murine macrophage, resulting in a predominantly anti-inflammatory effect of *L. rhamnosus* LRH30 and EPS.

## Introduction

A higher awareness of the relationship between diet and health has led to an increased demand for a new generation of food with enhanced and improved functionalities (Roberfroid 2002). A central strategy to achieve this is through the addition and utilization of probiotics and generally regarded as safe (GRAS) organisms, renowned for their ability to enhance food products and beverages through the growth and excretion of secondary metabolites (Sørensen, Rochfort, et al. 2023). An essential component in facilitating this production of functional food ingredients is bioprocess condition optimisation to maximise the yield of these valuable biocomponents. Design of experiments (DOE) is a systematic statistical optimisation approach with the advantage of reducing the number of experiments required while maintaining a robust experimental framework, rendering it ideal for parameter optimisation (Myers 1999).

Lactic acid bacteria (LAB) and bifidobacteria hold a significant status as probiotics, due to their highly beneficial impacts on human health, (Gorbach 1990). and include species of Lactobacillales and Bifidobacteriales, respectively.

In addition to their probiotic abilities, these microorganisms are also ubiquitous within the human diet across the world as essential constituents of fermented foods and beverages (Tamang et al. 2020) *Lacticaseibacillus rhamnosus* (*L. rhamnosus*) is one of the most prominent species of probiotic bacteria characterised, and *L. rhamnosus* GG was the first strain of *Lactobacillus* to be patented in 1989 due to its promising probiotic potential (Gorbach et al. 1989). Since then, strains belonging to *L. rhamnosus* have been widely applied in probiotic and functional foods across the world (Puebla-Barragan and Reid 2019). In addition to the probiotic potential of the species *L. rhamnosus* itself, it is also a well-known producer of exopolysaccharides (EPS) (Sørensen et al. 2022). EPS are natural biodegradable polymers excreted by microorganisms and play a crucial role in several biological processes including biofilm formation, protection against environmental stresses and can furthermore act as carbohydrate storage for the cells (Moscovici 2015; Ryan et al. 2015; Nguyen et al. 2020). Besides their physiological role, EPS has been recognised for conferring a multitude of health benefits including immunomodulatory, gut stimulatory and cholesterol-lowering activities (Sørensen et al. 2022).

The commercial interest of EPS lies within its multifaceted applications across several industries exemplifying the significance of this biopolymer. In the food industry, LAB-producing EPS has actively been applied in yoghurt production to prevent syneresis (London et al. 2015), has reduced moisture levels in reduced-fat cheese production (Perry et al. 1997; Dabour et al. 2006), has been shown to improve dough rheology and sensory properties of sour-dough products (Galle et al. 2010) and can be utilized as an emulsifier in food matrices (Saleem et al. 2021). Outside the food industry, EPS has also shown potential as a drug-delivery agent in the pharmaceutical industry (Dahiya and Nigam 2023) and as a plant protection agent in the agricultural industry (Blainski et al. 2018).

The total possible yields of both cells and EPS obtained from *L. rhamnosus* bioprocesses are dependent on several factors including media composition, pH, temperature and airflow (Sørensen et al. 2022). The media composition for optimal production of biomass in dairy-based substrates was previously optimised through a DOE approach, with skim milk powder resulting in the highest yields (Sørensen et al. 2024). Temperature is a fundamental environmental variable, and have in previous studies shown different optima for EPS and biomass production, with the optimal temperature for the growth of *L. rhamnosus* is reported to be at 37 °C (Wang et al. 2020a, b), the optimal temperature for EPS production is however reported between 20 °C and 25 °C (Gamar et al. 1997; Oleksy-Sobczak et al. 2020). The dependency of aeration *on L. rhamnosus* biomass and EPS has also been investigated previously, with the best results observed at partially anaerobic conditions (Gamar-Nourani et al. 1998).

The primary objective of this study is to systematically investigate the effects of temperature and airflow on the production of biomass and EPS from *L. rhamnosus* in batch bioprocesses. Leveraging the statistical optimisation of DOE through response surface methodology, the optimal conditions will be validated first in small-scale bioprocesses and later in scale-up. Finally, the immunomodulatory potential of both biomass and EPS is evaluated in vitro through their capability to influence the secretion of cytokines IL-1, IL-6, IL-10 and TNF-α and the chemokines MIP-2 and MCP in murine macrophages.

## Materials and methods

### Microorganism and inoculum preparation

*Lacticaseibacillus rhamnosus* LRH30 was supplied by Sacco System (Cadorago, Italy). 2 µL of stock culture of *L. rhamnosus* LRH30 was precultured in 100 mL sterile (121 °C for 20 min) de Man, Rogosa and Sharp (MRS) broth (Oxoid, United Kingdom), in a conical flask for 24 h at 37 °C and 100 rpm.

The inoculum for the bioreactor cultivations was prepared by collecting the cell pellet from the preculture by centrifugation for 5 min at 5000 g and then resuspended in DI water to a total volume of 10 mL. Inoculum was added to the bioreactors in a 2% cell concentration.

### Media preparation

The media was skim milk based with a salt solution derived from based basal minimal media (BMM) media (Macedo et al. 2002) and consisted of 37.05 g/L of skim milk powder (Dairygold, Ireland), 14 g/L yeast extract (Thermo Fischer Scientific, Massachusetts, USA), 5 g/L sodium acetate anhydrous (Thermo Fischer Scientific, Massachusetts, USA), 2 g/L ammonium citrate (Alfa Aesar, Massachusetts, USA), 3 g/L KH_2_PO_4_ (Thermo Fischer Scientific, Massachusetts, USA), 0.2 g/L MgSO_4_ (Honeywell, Indiana, USA), 0.04 g/L MnSO_4_ (Thermo Fischer Scientific, Massachusetts, USA), 0.034 g/L FeSO_4_ (Thermo Fischer Scientific, Massachusetts, USA), 0.5 g/L cysteine hydrochloride 1 g/L (Thermo Fischer Scientific, Massachusetts, USA), tween 80 (Thermo Fischer Scientific, Massachusetts, USA) and 2 ml/L of antifoam (Murphy & Son, Ireland). Media was autoclaved at 110 °C for 10 min to prevent the formation of milk gels that have been observed at higher autoclavation temperature.

### Bioreactor setup

Cultivations of *L. rhamnosus* for the DOE were conducted in a 500 mL bioreactor with a working volume of 250 mL (Bioxplorer 400, H.E.L Group, Hemel Hempstead, United Kingdom). The temperature was varied at a range of between 20 °C and 40 °C to accommodate optimal values described for both biomass and EPS production in literature. The airflow rate was varied between 0 and 0.83 vvm to both test growth under no or low oxygen as well as ensuring adequate oxygen transfer while avoiding foaming. The air was supplied through a sintered sparger. pH was controlled by the addition of either 3 M NaOH or 0.5 M H_2_SO_4_ using peristaltic pumps connected to a computer. Agitation was set to 200 rpm for complete homogenization of the broth. pH and dissolved oxygen (DO) were monitored through probes (Gettinge, Gothenburg, Sweden).

Scale-up cultivations of *L. rhamnosus* LRH30 at optimised conditions for biomass and EPS production were conducted in a 3.6 L bioreactor with a working volume of 2 L (KLF, BioEngineering AG, Wald, Switzerland). The temperature and airflow rate were kept constant throughout the processes. pH was maintained at 6 (± 0.2) by the addition of either 3 M NaOH or 0.5 M H_2_SO_4_ utilizing a peristaltic pump connected to the computer. Agitation was set at 200 rpm to ensure complete mixing of the broth. Temperature, pH and DO were monitored through probes (Mettler-Toledo, Columbus, USA) and controlled by software supplied by Bioengineering. Total cell density (TCD) was monitored through a NIR probe at 880 nm (Hamilton Company, Reno, United States).

In the scale-up process, it was decided to maintain constant parameters such as oxygen transfer coefficient (kLa), power per volume (P/V), and hydrodynamic conditions, as these factors were not expected to significantly influence the outcome for this specific strain. *L. rhamnosus* LRH30 is a facultative anaerobe with relatively low oxygen demands, and thus the oxygen transfer coefficient in the bioreactor did not require significant adjustment. As a result, we prioritized keeping temperature and airflow constant to minimize complexity while ensuring reproducibility. Further changes in kLa or P/V were considered unlikely to provide significant improvements in yield and were therefore maintained constant for this study. For all cultivations, inoculum was added to achieve a 2% concentration in the bioreactors.

### Experimental design

Response surface methodology was applied to optimise two factors: temperature and airflow. A central composite design was used with three repetitions in the centre to account for pure internal error and was designed to optimise the biomass and EPS yields. Each factor consisted of 5 levels and included the axial points (plus and minus 2), factorial points (plus and minus 1) and the centre points, leading to 12 cultivations in total (Table 1).

**Table 1:** Experimental range and coded levels of variables

Factors	Coded levels				
	− 2	− 1	0	1	2
Temperature (°C)	20	22.93	30	37.07	40
Airflow (vvm)	0	0.122	0.42	0.71	0.83

A quadratic polynomial equation was applied to correlate the process parameters to the response variables biomass or EPS:

1 $$Y= {\beta }_{0}+\sum {{\beta }_{i}X}_{i}+\sum {\beta }_{ij}{X}_{i}{X}_{j}\sum {\beta }_{ii}{X}_{i}^{2} ;i\ne j, i,j=\text{1,2},3,\dots k$$

where *Y* is either the biomass in g/L or EPS in mg/mL, *β*_*0*_ is the intercept, *β*_*i*_ is the linear relation, *β*_*ij*_ is the interaction term, *β*_*ii*_ is the quadratic term and *i* and *j* represent the process variables.

Regression analysis of the experimental data was performed using Design Expert 13.0.13.0 (Stat-Ease, Inc., Minneapolis, USA).

### Off-gas analysis

The respiratory quotient (RQ) was calculated based on the oxygen uptake rate (OUR) and the carbon dioxide evolution rate (CER). These were calculated post-fermentation by Eqs. 2 and 3:

2 $$OUR= \frac{\rho \bullet {M}_{O2}}{R\bullet T}\left({F}_{Air,in}\bullet \frac{{C}_{O2,cal}}{100}-{F}_{Air,out }\bullet \frac{{C}_{O2,out}}{100}\right)$$

3 $$CER= \frac{\rho \bullet {M}_{CO2}}{R\bullet T}\left({F}_{Air,out}\bullet \frac{{C}_{CO2,out}}{100}-{F}_{Air,in}\bullet \frac{{C}_{CO2,cal}}{100}\right)$$

From this, RQ could be calculated, Eq. 4:

4 $$\frac{CER}{OUR}$$

Where p is the pressure (bar), M_O2_ is the molecular weight of O_2_ (g/mol), F_Air,in_ is the airflow going into the bioreactor (mL/h), C_O2,cal_ is the concentration of O_2_ in the air (%), F_air,out_ is the measured airflow going out (mL/h), C_O2.out_ is the concentration of the measured of O_2_ (%), M_CO2_ is the molecular weight of CO_2_ (g/mol), C_CO2.out_ is the concentration of the measured of CO_2_ (%) and C_CO2,cal_ is the concentration of CO_2_ in the air (%).

### Cell dry weight determinations

Samples from the bioprocess were collected through the cultivations and 2 mL of bacterial cell suspensions were centrifuged at 3360×*g* for 10 min. Cell pellets were washed twice in distilled water and dried overnight at 80 °C. The dried cell pellet was then weighed to determine the bacterial CDW.

### Exopolysaccharide isolation and quantification

At the end of cultivation, the broth was harvested and cells were removed by centrifugation for 15 min at 10,000×*g*. After cell removal, residual protein in the media was precipitated with 10% final volume of trichloroacetic acid (Thermo Fischer Scientific, Massachusetts, USA) and subsequently removed by centrifugation for 15 min at 10000×*g*. The crude EPS was then precipitated by the addition of cold 95% ethanol in a 1:2 volume and left at 4 °C for 48 h. The supernatant was discarded and crude EPS were mixed with an equal volume of deionised (DI) and subjected to dialysis for 48 h using a 12–14 kDa membrane (Merck, Darmstadt, Germany) to remove low molecular weight impurities, residual ethanol and salts. Finally, the sample was lyophilised for 48 h to obtain a powder.

The EPS content in the powder was analysed for total carbohydrates by the phenol–sulphuric method with glucose as standard (Dubois et al. 1956).

### Fourier-transform infrared spectroscopy

The infrared spectra of the purified EPS powder were acquired using a Perkin Elmer Spectrum Two FTIR spectrometer (Perkin Elmer, Massachusetts, United States) equipped with a diamond universal attenuated total reflectance accessory. The sample was placed on the crystal for measurement in the mid-infrared region, covering frequencies in the range of 4000–400 cm^−1^.

### Growth of J774A.1 murine macrophage cell stocks

Stocks of J744A.1 murine macrophage (J774) was maintained and grown as previously described (Sørensen et al. 2023). A volume of 250 µL of J774 macrophage cells was loaded on a 96-well microtiter plate in 10^6^ cells/mL concentration before being incubated at 37 °C in a humidified, 5% CO_2_ atmosphere for 24 h.

A 96-well microtiter plate (Thermo Fisher Scientific, Massachusetts, USA) was loaded with 250 µL of J774 cells in a concentration of 10^6^ cells/mL which were then incubated at 37 °C in a humidified, 5% CO_2_ atmosphere for 24 h. Samples were added in a concentration of 25 mg/mL for skim milk powder (SMP), *L. rhamnosus* LRH30 and broth while EPS was added in a concentration of 25 μg/mL.

### Enzyme-linked immunosorbent assay (ELISA) and MTS cell viability assay

The concentration of the secretion of the cytokines interleukin (IL)-1β, IL-6, IL-10, tumour necrosis factor (TNF)-α, macrophage inflammatory protein (MIP)-1, MIP-2, and monocyte chemoattractant protein (MCP)-1 were determined by sandwich ELISA and a MTS cell viability assay was carried out as previously described (Sørensen et al. 2023).

## Results

### Response surface and central composite design methodology for optimisation

The optimisation of the process parameters temperature and airflow was carried out by employing a statistical approach for maximum biomass and EPS production respectively through a central composite design (CCD) in the BioXplorer setup.

The matrix consisted of 12 experimental runs that were randomised to minimise the possibility of bias. Table 2 presents an overview of the experimental and predicted values from the 12 experiment. The yield of biomass produced by *L. rhamnosus* LRH30 varied between 6.4 and 9.7 g/L while the EPS yields varied between 171.5 and 366.4 mg/L.

**Table 2:** Coded experimental design and results for RSM of maximum biomass and EPS X1: Temperature (°C), X2: Airflow (vvm)

Runs	Coded levels		Biomass production (g/L)		EPS production (mg/L)	
	X_1_	X_2_	Experimental	Predicted	Experimental	Predicted
1	+ 1	+ 1	9.5	9.6	178.5	179.5
2	0	+ 2	8.9	8.9	201.1	194.4
3	0	0	9.0	8.9	248.6	254.7
4	0	0	8.9	8.9	266.5	254.7
5	− 2	0	6.3	-	373.5	350.7
6	− 1	+ 1	7.1	7.1	366.4	387.6
7	+ 1	+ 1	9.4	9.3	338.2	337.0
8	+ 2	+ 2	9.7	9.7	335.0	337.0
9	− 1	− 1	6.4	6.4	158.6	165.7
10	0	− 2	8.0	8.0	171.5	167.3
11	0	0	9.0	9.0	253.2	254.7
12	0	0	9.0	9.0	247.2	254

The model that describes the data most accurately was found by conducting an analysis of variance (ANOVA) with backwards regression to evaluate all the model terms. From this, a full quadratic equation was found to be the best fit by evaluating the F-values for both biomass and EPS production (Table 3 and Table 4). In the model development, it was discovered that including all factorial points resulted in a model for predicting the response variable biomass with a significant lack of fit. Upon examination, one data point characterized by the -2 variable for temperature and 0 variable for airflow exhibited anomalous behaviour. Post-analysis diagnostic plots identified this point as an outlier. To ensure a robust and statistically valid model, this point was excluded after careful consideration of its impact on model integrity.

**Table 3:** Analysis of variance (ANOVA) for the quadratic model of biomass production for *L. rhamnosus* LRH30 from the experimental results

	Sum of squares	df	Mean square	F-value	p-value	
Model	0.17	5	0.034	474.5	< 0.0001	Significant
A-air flow	0.009	1	0.01	130.7	< 0.0001	
B-temperature	0.14	1	0.14	1943.7	< 0.0001	
AB	0.002	1	0.002	21.1	0.0059	
A^2^	0.006	1	0.006	87.7	0.0002	
B^2^	0.03	1	0.028	393.3	< 0.0001	
Residual	0.0004	5	0.0001			
Lack of fit	0.0003	2	0.0001	4.8	0.12	Not significant

*df* degrees of freedom

**Table 4:** Analysis of variance (ANOVA) for the reduced quadratic model of EPS production from* L rhamnosus LRH30* from the experimental results

	Sum of squares	df	Mean square	F-value	p-value	
Model	0.98	5	0.2	86.3	< 0.0001	significant
A-air flow	0.02	1	0.02	9.82	0.0202	
B-temperature	0.002	1	0.002	0.73	0.4260	
AB	0.54	1	0.54	238.6	< 0.0001	
A^2^	0.18	1	0.18	79.8	0.0001	
B^2^	0.15	1	0.15	66.0	0.0002	
Residual	0.014	6	0.002			
Lack of fit	0.010	3	0.003	2.8	0.2117	Not significant

*df* degrees of freedom

Both models were statistically significant with an F-value of 474.5 for biomass predictions and an F-value of 86.3 for EPS prediction, with only a 0.01% chance that these high F-values occur due to noise. Both models additionally had p-values of < 0.0001 further deeming them significant.

The adjusted R^2^ value were 0.99 for the prediction of biomass meaning that less than 1% of the variability in the data is due to noise, which is further supported by the lack of fit that had an F-value of 0.12 deeming it insignificant. The predicted R^2^ for this model was 0.97 indicating good linearity between the predicted and actual values.

For the response variable biomass, all variables individually, their interactive and quadratic terms all significantly contribute to the model with p-values of less than 0.05 (Table 3).

The adjusted R^2^ value for the prediction of EPS was 0.96, indicating that 96% of the variability in the response is accounted for in the model, leaving just 4% assignable to noise. The predicted R^2^ value was 0.92 which shows that this model did have a strong predictive power. The lack of fit for this model was insignificant with an F-value of 0.21. For the response variable EPS, only airflow individually was significant for the model with a p-value of 0.02. In contrast, temperature alone did not significantly contribute to EPS production with a p-value of 0.42 (Table 4). However, both the interactive terms as well as the quadratic terms significantly contributed to the model indicating that the effect of temperature has a non-linear relationship with EPS production but is also dependent on the airflow.

**Fig. 1:**
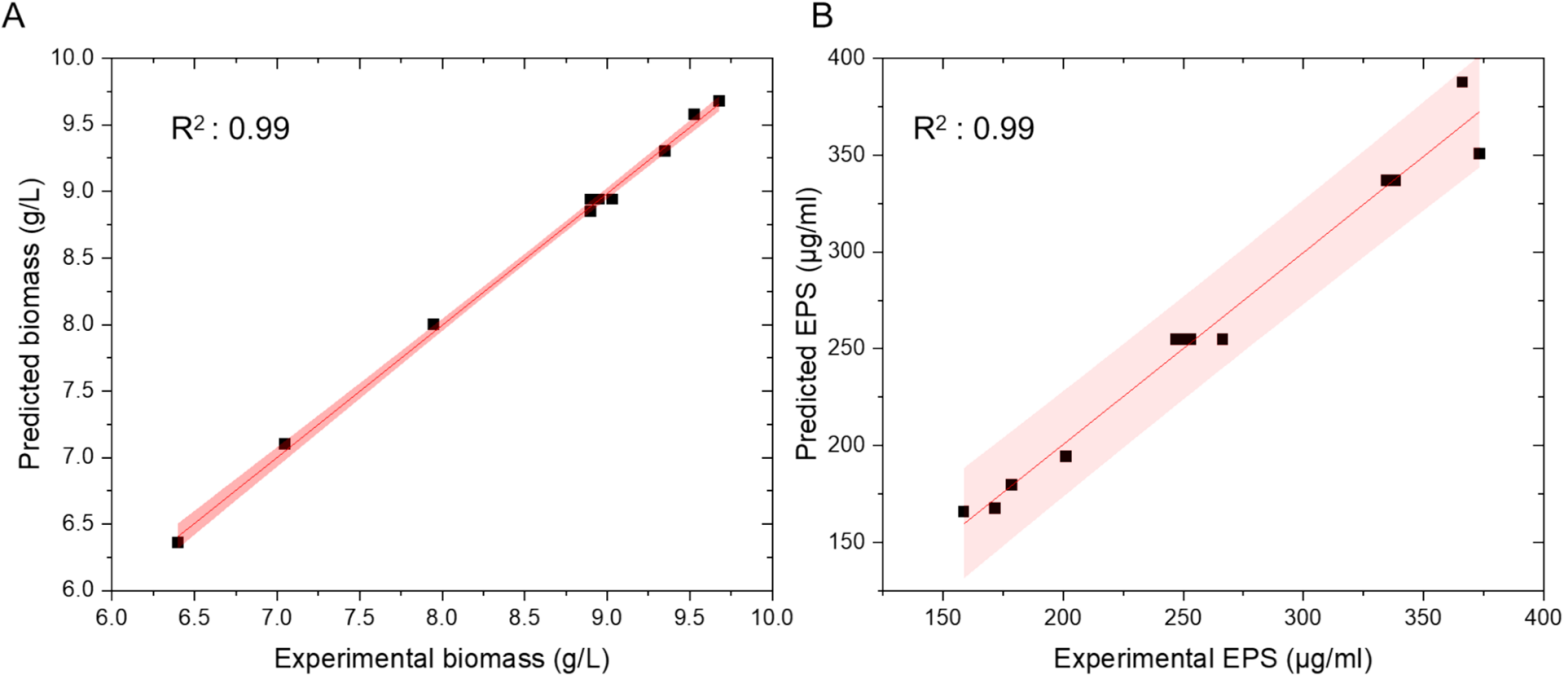
Comparison of actual vs predicted responses of **A** biomass and **B** EPS. 95% confidence intervals are highlighted in red

The comparison of actual biomass values against predicted biomass can be seen in Fig. 1A. There is a good linearity between the values, signified by the high R^2^ of 0.99 as well as a narrow confidence interval underlining the highly predictive power of this model. The examination of the relation between experimental and predicted values of EPS is shown in Fig. 1B. Here, the regression analysis again showed a good linear fit with an R^2^ of 0.99 indicating that also this model is powerful in predicting EPS values. Despite slightly wider confidence intervals than those observed for biomass, the predictive power remains evident.

The responses were analysed by multiple regression analysis and the final coded equations for predicting biomass (Eq. 5) and EPS (Eq. 6):

5 $$\text{ln}\left(biomass\right)=2.19+0.03\times A+0.17\times B-0.02\times AB-0.03\times {A}^{2}-0.08\times {B}^{2}$$

6 $$\text{ln}\left(EPS\right)=5.54+0.05\times A-0.01\times B-0.37\times AB-0.17\times {A}^{2}+0.15\times {B}^{2}$$

where *A* is the airflow (vvm) and *B* is the temperature (°C).

### Optimisation of bioprocess conditions

Three-dimensional contour plots were drawn for the visualization of the interactions between the two variables in each of the responses.

**Fig. 2:**
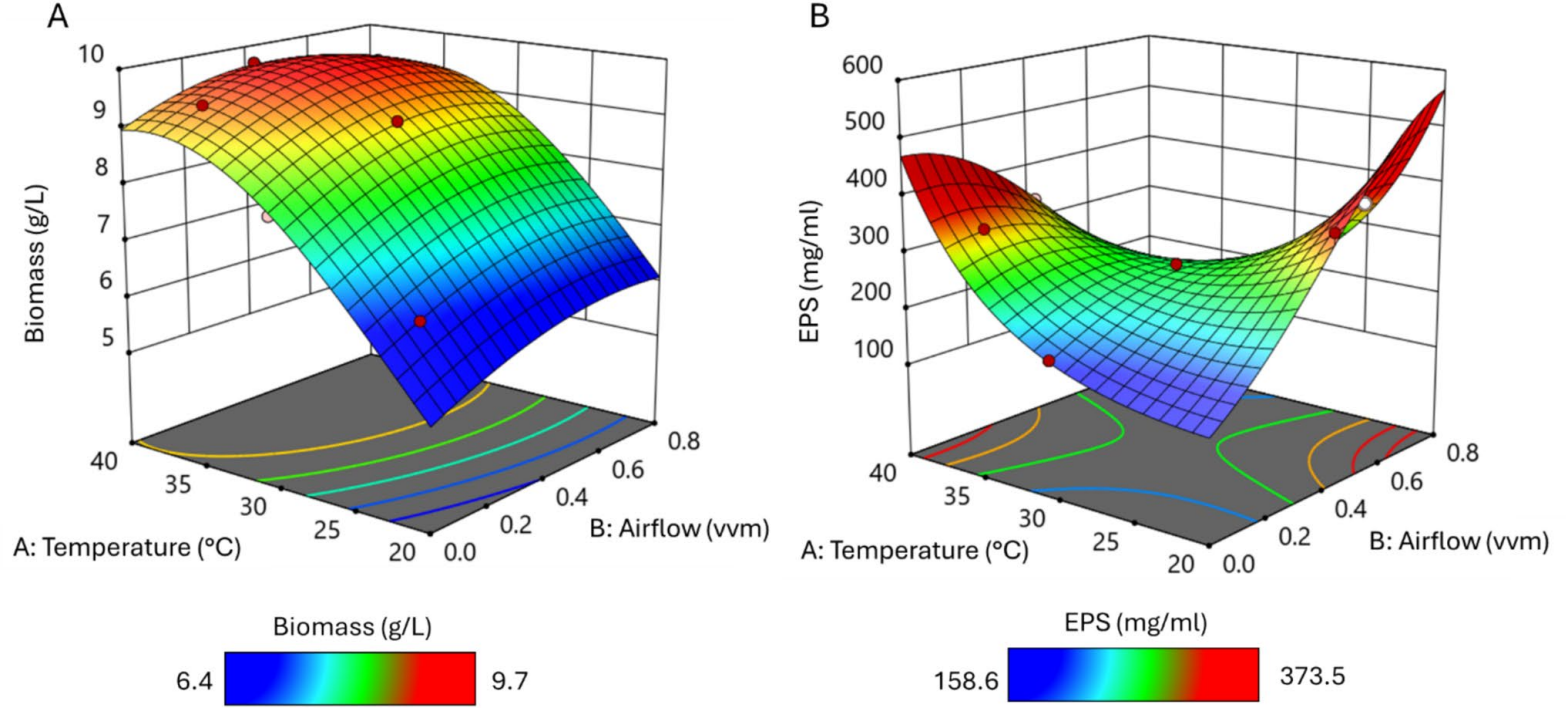
3D contour plots of process parameter optimisations.** A** Interaction between A: airflow (vvm) and B: temperature (°C) Biomass (g/L) as the response variable and** B** EPS (mg/mL) as the response variable

The contour plot showing the optimal conditions for biomass production is shown in Fig. 2A. The curvature of the plot shows that the optimal temperature for cell growth is at higher temperatures, while the effect of airflow (vvm) appears to be less important as high values of biomass are always reached with a high temperature. By analysing and solving the 3D contour plots the optimal conditions for high predicted biomass yields of 9.7 g/L were deemed to be at 37.1 °C with an airflow rate of 0.46 vvm.

The 3D contour plot for optimisation of EPS production is shown in Fig. 2B. There appear to be two local optima for temperature as seen by the curvature of the plot, that is simultaneously dependent on the airflow. Solving the plot, however, reveals that the highest predicted yield of EPS of 556 mg/mL can be reached at a temperature of 20.1 °C with an airflow of 0.71 vvm.

### Bioprocess data analysis

The data from the 12 bioprocesses was evaluated in terms of base consumption and respiratory quotient (RQ) as indicators for metabolic activity as well as dissolved oxygen (DO) due to the different levels of airflow.

**Fig. 3:**
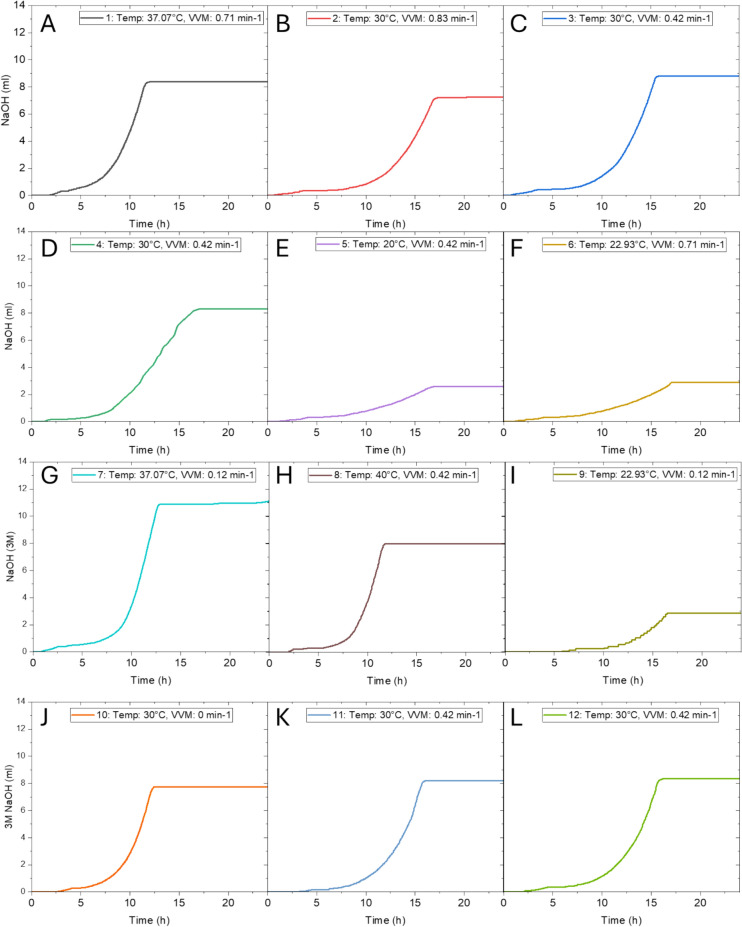
Base consumption profiles of the 12 bioprocess runs. **A** Temperature 37.07 °C, airflow 0.71 min^−1^ **B** Temperature 30 °C, airflow 0.83 min^−1^, **C** Temperature 30 °C, airflow 0.42 min^−1^, **D** Temperature 30 °C, airflow 0.42 min^−1^, **E** Temperature 20 °C, airflow 0.42 min^−1^, **F** Temperature 22.93 °C, airflow 0.71 min^−1^, **G** Temperature 40 °C, airflow 0.42 min^−1^, **H** Temperature 40 °C, airflow 0.42 min^−1^, **I** Temperature 22.93 °C, airflow 0.12 min^−1^, **J** Temperature 30 °C, airflow 0 min^−1^, **K** Temperature 30 °C, airflow 0.42 min^−1^, **L** Temperature 30 °C, airflow 0.42 min^−1^

The pH of all experiments was controlled and maintained at 6. The addition of 3 M NaOH varied across the 12 processes between 2.84 and 11.09 mL (Fig. 1). The correlation between cell growth and temperature observed from the DOE becomes evident in the base consumption data, as all experiments with lower temperatures (Fig. 3E, F and I) had the lowest values of cell growth and the lowest values for addition of 3 M NaOH. Although these experiments yielded the lowest biomass, they simultaneously also yielded the highest amounts EPS. The airflow also does not appear to influence the biomass, as the process with no airflow and high airflow at the temperature centre point of 30 °C (Fig. 3B and I) have values for additions of base that are close of 8.39 and 7.72 mL respectively. This is further confirmed by the biomass yield of 8.9 g/L which is identical in these two experiments (Table 2).

**Fig. 4:**
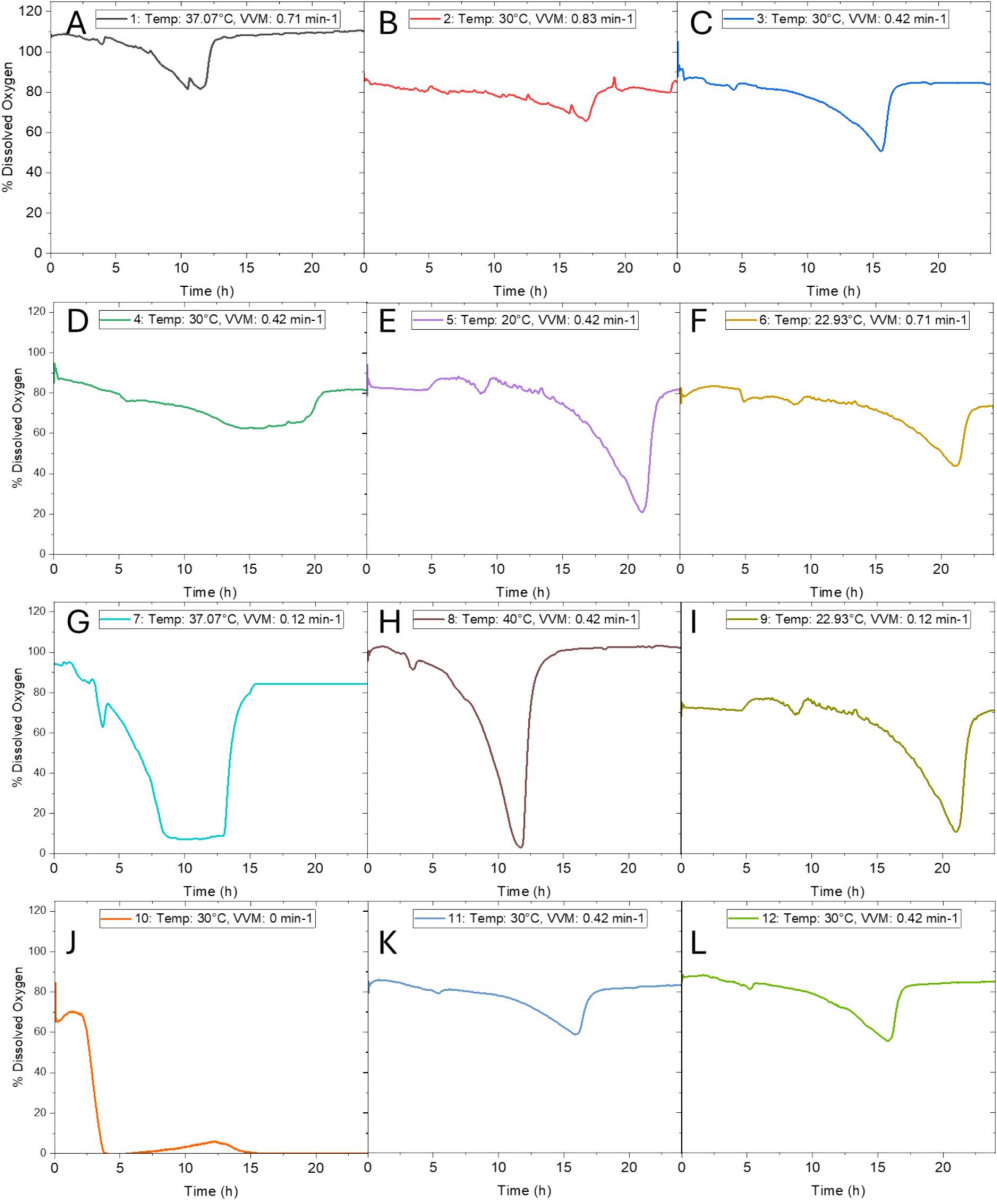
DO profiles of the 12 bioprocess runs. **A** Temperature 37 °C, airflow 0.71 min^−1^ **B** Temperature 30 °C, airflow 0.83 min^−1^, **C** Temperature 30 °C, airflow 0.42 min^−1^, **D** Temperature 30 °C, airflow 0.42 min^−1^, **E** Temperature 20 °C, airflow 0.42 min^−1^, **F** Temperature 22.9 °C, airflow 0.71 min^−1^, **G** Temperature 40 °C, airflow 0.42 min^−1^, **H** Temperature 40 °C, airflow 0.42 min^−1^, **I** Temperature 22.9 °C, airflow 0.12 min^−1^, **J** Temperature 30 °C, airflow 0 min^−1^, **K** Temperature 30 °C, airflow 0.42 min^−1^, **L** Temperature 30 °C, airflow 0.42 min^−1^

Figure 4 shows the DO profiles for the 12 experiments (labelled as A-L). Airflow was varied between all experiments from 0 vvm to 1.24 vvm and controlled through a sintered sparger. The DO was continuously monitored and is of interest to investigate due to the facultative anaerobe nature of *L. rhamnosus*. The levels of DO fell during all cultivations, clearly indicating the consumption of oxygen by the cells. There appeared to be a pattern between the high EPS production at low temperatures would only occur if the airflow was simultaneously kept high and at the experimental run without supplied oxygen, the EPS production was correspondingly low at 171.54 mg/L, Fig. 4J.

**Fig. 5:**
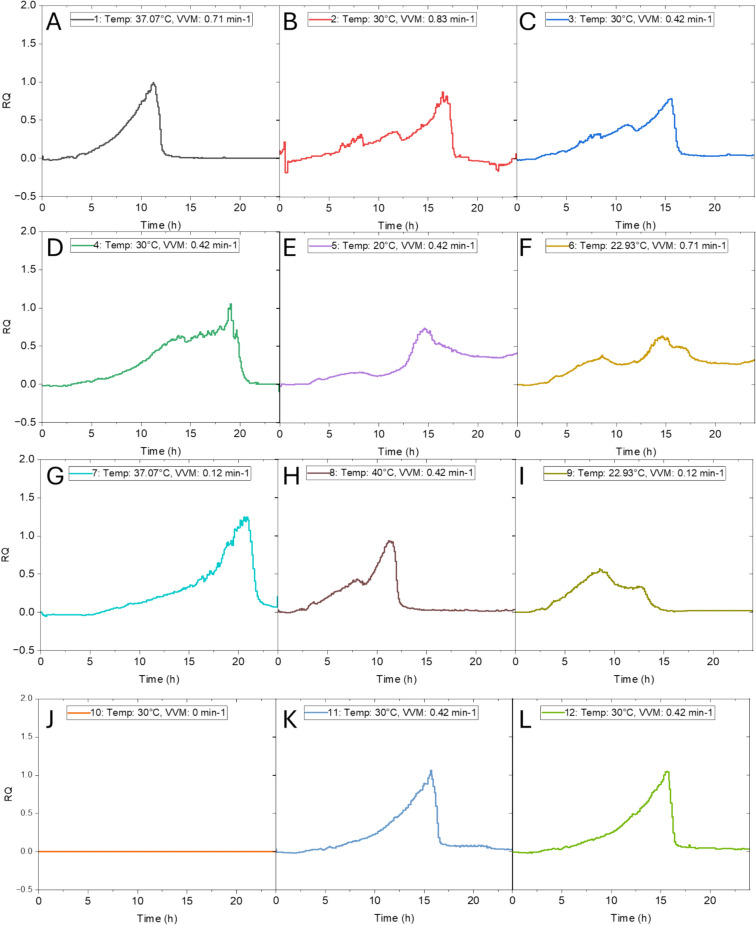
Respiratory quotient profiles of the 12 bioprocess runs. **A** Temperature 37.07 °C, airflow 0.71 min^−1^ **B** Temperature 30 °C, airflow 0.83 min^−1^, **C** Temperature 30 °C, airflow 0.42 min^−1^, **D** Temperature 30 °C, airflow 0.42 min^−1^, **E** Temperature 20 °C, airflow 0.42 min^−1^, **F** Temperature 22.93 °C, airflow 0.71 min^−1^, **G** Temperature 40 °C, airflow 0.42 min^−1^, **H** Temperature 40 °C, airflow 0.42 min^−1^, **I** Temperature 22.93 °C, airflow 0.12 min^−1^, **J** Temperature 30 °C, airflow 0 min^−1^, **K** Temperature 30 °C, airflow 0.42 min^−1^, **L** Temperature 30 °C, airflow 0.42 min^−1^

The RQ of the experiments were calculated based on gas analysis that was continuously monitored during the bioprocesses and the calculated values vary between 0 and 1.2. As airflow is a parameter used in the calculation for RQ, the data seen in Fig. 5J which had no addition of our is equal to 0. The RQ reaches a peak in all experiments before gradually decreasing, however the magnitude of the peak differs between experiments. The increase in RQ reflects the cell growth, and the intensity of the peak reflects the effectiveness in substrate utilization by *L. rhamnosus* LRH30. Consistent with earlier observations, the experiments conducted at lower temperatures exhibit distinct features, here characterised by less prominent peaks in the RQ plots. This trend is following the previous findings, where these experiments also yielded the lowest amounts of biomass (Fig. 5E, F and I).

### Validation of model in BioXplorer system

To validate the optimal conditions identified by the CCD optimisation, experiments with the optimal conditions were set up and performed in triplicates (Table 5) in the BioXplorer system. The bioprocesses yielded results that were consistent and reproducible across the triplicates for both of the optima. The results yielded from this experimental evaluation of were similar to the predicted optimal values predicted by the two models. The predicted biomass was 9.7 g/L while the experimental values were slightly higher at 10.1 g/L ± 2. This slightly exceeds the upper prediction interval, reflecting that the variability is not fully accounted for in the current model, but the model still demonstrates overall predictive accuracy for process optimisation."The predicted yield of EPS was 556 mg/L and here the experimentally obtained value was lower with a yield of 520.5 ± 24 mg/L. The similarity between the response and the predictions highlights the model's capability to accurately predict the optimal conditions, thereby facilitating the optimisation of the bioprocess for improved productivity.

**Table 5:** Optimal conditions for highest yields of biomass and EPS from *L. rhamnosus* LRH30 as identified by the CCD

	Temperature (°C)	Airflow (vvm)	Response	Predicted
Biomass	37.01	0.12	10.1 ± 2 g/L	9.7 g/L
EPS	20.1	0.18	520.5 ± 24 mg/L	556 mg/L

**Fig. 6:**
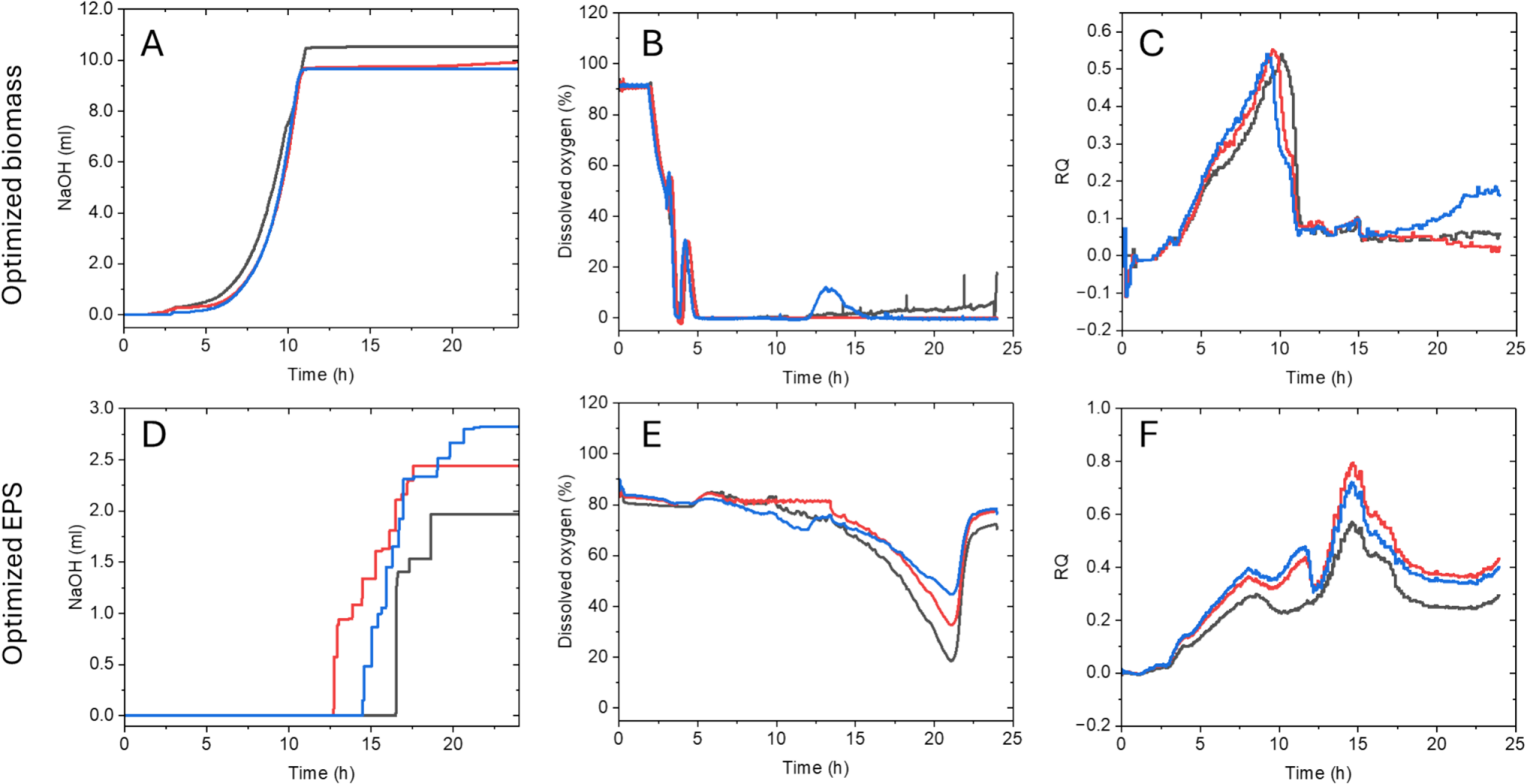
Bioprocess analysis of optimum conditions for biomass and EPS production from *L. rhamnosus* in triplicate experiments. **A** Base consumption for optimised biomass experiments. **B** DO for optimised biomass experiments, **C** RQ for optimised biomass experiments, **D** Base consumption for optimised EPS experiments. **E** DO for optimised EPS experiments, **F** RQ for optimised EPS mass experiments

The bioprocess data from the optimisation experiments can be seen in Fig. 6. The base consumption data from the biomass experiments (Fig. 6A) depicts an exponential growth curve and is in accordance with the high biomass yielded across the triplicate experiments. It can be observed that the addition of base ceases just after 10 h, which aligns with the decrease of the peak observed in the RQ data (Fig. 6C) and can be assumed to be the end of the growth phase. The DO data drops to zero shortly after 5 h (Fig. 6B), but this does not appear to affect cell growth, once again underscoring the facultative anaerobe nature of *L. rhamnosus* LRH30. In the optimisation experiments assessing EPS production, the base consumption (Fig. 6D) and DO plots (Fig. 6E) reveal a subdued metabolic activity in the bioreactor during the initial 10 h, apparent by no addition of base as well as a constantly maintained DO value at around 80%. While the RQ plot exhibits some activity in the earlier hours of the process, the majority of the microbial activity happens between 10 and 15 h, indicated by the modest peak in RQ observed in this time window.

### Validation of model in scale-up

The initial bioprocess DOE was conducted in a 250 mL bioprocess setup with the aim to optimise the temperature and airflow for improved biomass and EPS production. These optimised conditions were now scaled up and implemented in a 3.6 L bioreactor, running with a 2 L bioprocess to assess the scalability and performance of these processes on a larger scale. In addition to measuring base consumption, DO and gas analysis, these processes were also equipped with a Total Cell Density (TCD) probe.

**Fig. 7:**
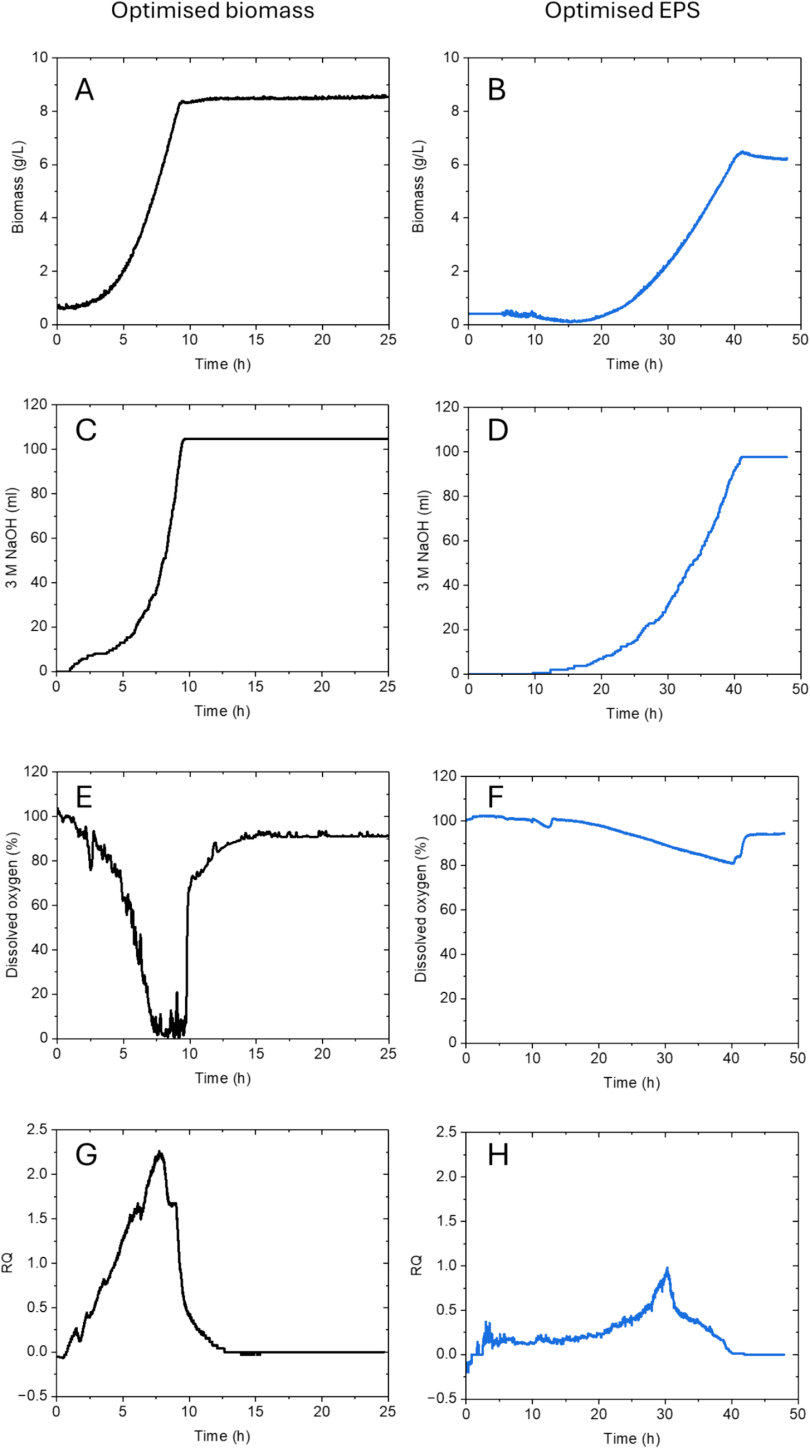
Bioprocess analysis of optimum conditions for biomass and EPS production from *L. rhamnosus* in a 2 L bioreactor. **A** Biomass for optimised biomass bioprocess. **B** Biomass for optimised EPS bioprocess **C** Base consumption for optimised biomass bioprocess **D** Base consumption for optimised EPS bioprocess **E** DO for optimised biomass bioprocess, **F** DO for optimised EPS bioprocess **G** RQ for optimised biomass bioprocess **H** RQ for optimised EPS mass bioprocess

Previously, the time course for the optimised conditions was synchronised at 24 h. However, for the scale-up experiments, real-time monitoring of biomass production was available from a TCD probe and facilitated adjustments to the setup, allowing the processes to progress until the stationary phase. This was at 24 h for the conditions optimised for biomass and 48 h for conditions optimised for EPS production. The live TCD probe revealed that the maximum biomass obtained was 8.54 g/L in experiments optimised for biomass (Fig. 7A), which was lower than the biomass obtained in the 250 mL setup. The maximum biomass yielded in conditions optimised for EPS was 6.25 g/L (Fig. 7B) The base consumption for the two processes showed an end of process sooner in conditions optimised for biomass than for EPS (Fig. 7C and D). Nevertheless, the total volume of base added was comparable for both processes, measuring values of 105 mL and 97 mL respectively. Differential airflow rates as seen in Table 5 influenced the DO profiles. The biomass-optimised process was operated with an airflow rate of 0.12 vvm while it was 0.18 vvm for the EPS-optimised process. Analysis of the DO profiles revealed different patterns: the biomass-optimised process experienced a drop of DO to 0 during the exponential phase followed by an increase back to 100% during the log phase (Fig. 7E). The EPS-optimised process dropped to 80% during the exponential phase, before reaching 100% again during the log phase (Fig. 7F). Lastly, the RQ profiles furthermore showed how the process optimised for biomass production reached an exponential phase at an earlier stage than the process optimised for EPS production (Fig. 7G and H). Following the optimisation runs in scale-up, quantification of EPS was conducted and was found to be 654 mg/L in the process optimised for EPS, with the addition of a longer process time of 48 h (Table 6).

**Table 6:** Optimal conditions for highest yields of biomass and EPS from *L. rhamnosus* LRH30 as identified by the CCD

	Temperature (°C)	Airflow (vvm)	Predicted	Response	
				250 mL batch	2 L batch
Biomass	37.01	0.12	9.7 g/L	10.1 ± 2 g/L	8.54 g/L
EPS	20.1	0.18	556 mg/L	520.5 ± 24 mg/L	654 mg/L

### Characterisation of EPS produced from L. *rhamnosus* LRH30 through FTIR

**Fig. 8:**
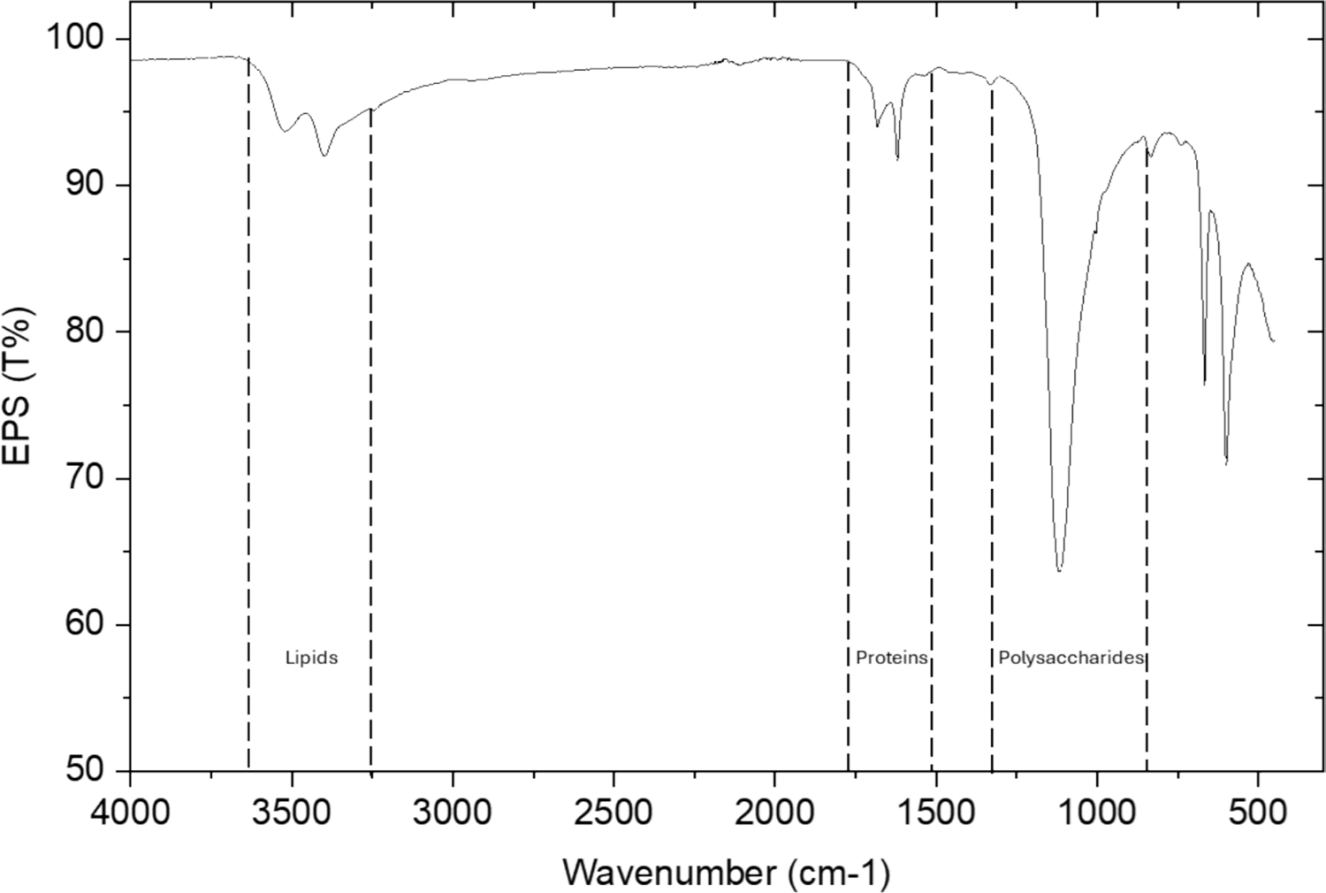
FTIR spectra of the produced EPS. The regions corresponding to lipids, proteins and polysaccharides are highlighted

FTIR analysis was performed to characterise the composition of the EPS produced. The spectra were obtained in the mid-infrared range (4000–400 cm^−1^) and can be seen in Fig. 8. The peaks were assigned to functional groups identified in other studies (Wang et al. 2008; Liang et al. 2010; Tang et al. 2017; Rehman et al. 2021).

The broad small spectrum band observed between 3300 and 3600 are the result of stretching vibrations of both hydroxyl and amino groups from carbohydrates and proteins (Liang et al. 2010). A small peak can also be observed in the range between 1500 and 1750 cm^−1^ and corresponds to the presence of stretching vibrations of CO, C=O and C–N bonds commonly found in proteins (Rehman et al. 2021). The large peak ranging at 800–1250 cm^−^1 demonstrates the stretching vibration on C–O–C and C–O typically associated with the presence of polysaccharides (Wang et al. 2008; Tang et al. 2017).

### Immunomodulatory activity of L. *rhamnosus* LRH30 and EPS

The immunomodulatory potential of the SMP, broth and *L. rhamnosus* LRH30 in doses of 25 mg/mL and EPS in a 25 μg/mL were assessed through MTS assay for macrophage viability and through ELISA for the secretion of cytokines IL-1β, IL-6, IL-10, TNF-α and chemokines MIP-1α/CCL3, MIP-2/CXCL2 and MCP.

**Fig. 9:**
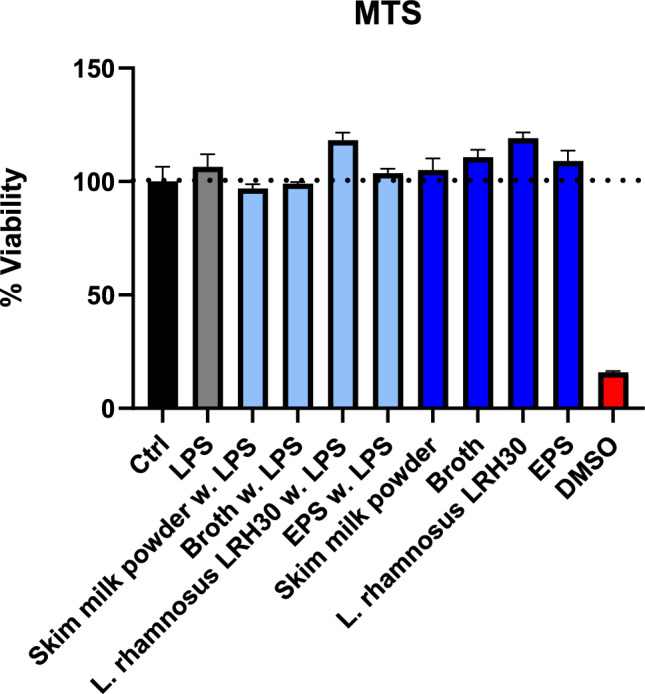
J774 viability after treatment with skim milk powder, broth, *L. rhamnosus* LRH30 in a 25 mg/mL dose as well as EPS in a 25 μg/mL dose with and without lipopolysaccharide (LPS) stimulation. DMSO was included as a negative control while LPS acted as a positive control. Error bars ± SEM, n = 3

The viability of macrophages in response to the different treatments can be seen in Fig. 9. When treated with LPS, none of the samples tested had a negative effect on the macrophage viability and stimulation *L. rhamnosus* LRH30 appeared to have a proliferative effect on the macrophages reaching a value of 118.23%. Similarly, in the absence of LPS, all samples demonstrated a slight proliferative effect with *L. rhamnosus* LRH30 again having the highest proliferative effect reaching 119.12%.

**Fig. 10:**
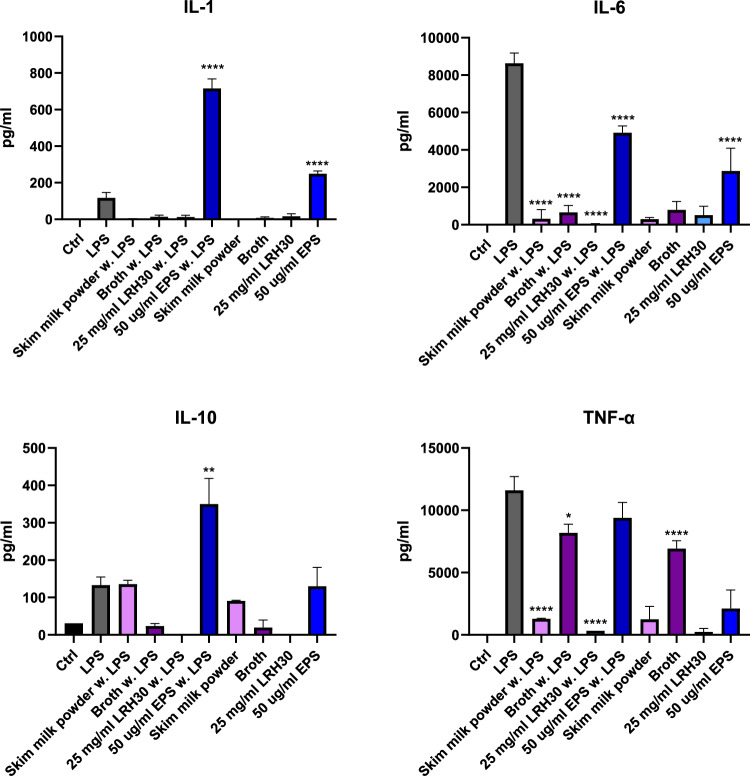
Secretion of IL-1β, IL-6, Il-10 and TNF-α by cells after treatment with skim milk powder, broth, *L. rhamnosus* LRH30 in a 25 mg/mL dose as well as EPS in a 50 μg/mL dose with and without lipopolysaccharide (LPS) stimulation. Significance p < 0.05 *, p < 0.01 **, p < 0.001 *** p < 0.0001 ****

**Fig. 11:**
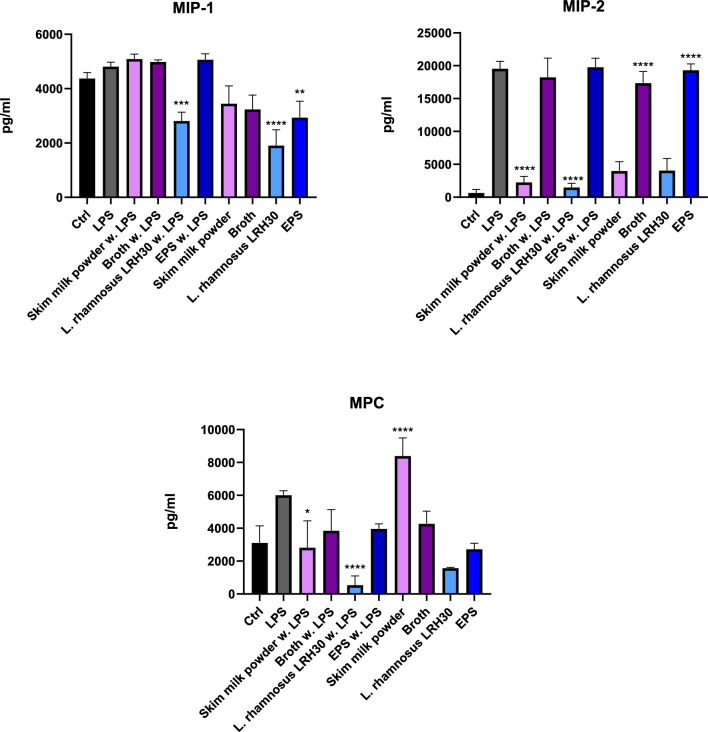
Secretion MIP-1α/CCL3, MIP-2/CXCL2 and MCP by cells after treatment with skim milk powder, broth and *L. rhamnosus* LRH30 in a 25 mg/mL dose as well as EPS in a 25 μg/mL dose with and without lipopolysaccharide (LPS) stimulation. Significance p < 0.05 *, p < 0.01 **, p < 0.001 *** p < 0.0001 ****

The immunomodulatory effect of SMP, fermentation broth, *L. rhamnosus* LRH30 and EPS was furthermore assessed through cytokine secretion (Fig. 10) and chemokine secretion (Fig. 11).

The cytokine excretion profiles of IL-1β, IL-6, IL-10 and TNF-α were examined in response to stimulation of the samples and LPS. The J774 macrophages secreted quite low levels of IL-1β, however, EPS did significantly increase the IL-1β secretion when treated with LPS. Both SMP, fermentation broth, *L. rhamnosus* LRH30 and EPS were capable of significantly reducing the secretion of IL-6 when stimulated with LPS. Broth and *L. rhamnosus* LRH30 significantly reduced the secretion of IL-10 in the presence of LPS while macrophages simulated with EPS and LPS had a significant increase in IL-10 secretion. Finally, both SMP and *L. rhamnosus* LRH30 significantly reduced the secretion of TNF-α when stimulated with LPS while a significant increase in TNF-α was observed in macrophages treated with fermentation broth alone (Fig. 10).

Finally, the secretion profiles of the chemokines MIP-1α/CCL3, MIP-2/CXCL2 and MCP by J774 macrophages were examined in response to treatment with SMP, fermentation broth, *L*. rhamnosus LRH30 and EPS. As for the cytokine secretion profiles, this was done with or without LPS. A significant reduction of MIP-1α/CCL3 was observed when treated with *L. rhamnosus* LRH30, both with and without LPS, while a reduction of MIP-1α/CCL3 secretion when treated with EPS was only observed in the absence of LPS. MIP-2/CXCL2 secretion however was significantly reduced both by treatment with SMP and *L. rhamnosus* LRH30 samples in the presence of LPS, while secretion of MIP-2/CXCL2 was significantly increased in macrophages alone when treated with fermentation broth and EPS. The secretion of MCP was significantly reduced when treated with *L. rhamnosus* LRH30 and stimulated with LPS, while SMP significantly increased the MCP secretion on macrophages alone (Fig. 11).

## Discussion

Exploring the influence of process parameters on the products of microbial bioprocesses offers opportunities for both biotechnology and functional food production.

This study aimed to optimise the important process conditions temperature and airflow in a small-scale bioreactor through a CCD setup to maximise the production of biomass and EPS by *L. rhamnosus* LRH30. The EPS was furthermore characterised through FTIR analysis and the immunomodulatory potential of both biomass, EPS and fermentation broth was assessed through ELISA.

The optimisation of process conditions through DOE for a bioprocess in skim milk-based media producing biomass and EPS from *L. rhamnose* LRH30 resulted in two different optima. For a bioprocess optimised for maximum biomass yield, the optimal conditions were revealed to be at 37.01 °C and 0.12 vvm. The predicted yield in the small-scale setup under these conditions was 9.7 g/L while the response was slightly higher at 10.1 g/L. Other studies have evaluated the effect of temperature on the growth of *L. rhamnosus* in dairy-based media with findings on growth optimum consistent with those of this study. One study tested a range of temperatures from 6 to 41 °C in milk-based media and the highest CFU counts at 35 °C (Medve and Lipt 2008) while another study cultivated *L. rhamnosus* in a whey-based substrate and determined the optimum temperature to be 37 °C (Wang et al. 2020a, b). The effect of aeration on *L. rhamnosus* growth has been investigated as well, and it was found that anaerobic conditions were not favourable for growth (Wang et al. 2020a, b). The bioprocess conditions for optimised EPS yield were determined to be at 20 °C and 0.18 vvm. It is however important to note that 20 °C represents the lowest temperature tested within the experimental range and further investigation at lower temperatures, outside of the current test space, may offer additional insights into the effect of temperature on EPS production. Other studies have also observed that lower temperatures of 25 °C (Oleksy-Sobczak et al. 2020) yielded the highest amounts of EPS from *L. rhamnosus* LRH30. The discrepancy between the optimal growth temperatures for biomass and EPS from L. rhamnosus LRH30 respectively has been applied in bioprocesses in another study by creating a temperature shift from the optimum growth temperature (37 °C) to optimum EPS temperature (25 °C) at the beginning of the exponential phase (Gamar-Nourani, Blondeau and Simonet, 1998). Here, it was observed that by shifting the temperature from optimum growth temperature at 37 °C to optimum EPS secretion temperature at 25 °C, the EPS yield was increased by 19%. This higher yield demonstrates a potential for capitalizing on both temperature optima in further development of batch and fed-batch bioprocess development.

The optimum conditions identified by the DOE in the 250 mL scale bioreactor were implemented in a scale-up setup in a 2 L bioprocess. In the scale-up of bioprocesses, one of the primary challenges lies in the consistency of product quality across the different scales. The process optimised for *L. rhamnosus* biomass at 37 °C and 0.12 vvm yielded 8.54 g/L of biomass in the scale-up setup, which was lower than the 10.1 g/L yielded in the 250 mL setup, but still within the statistical error. The loss of performance, productivity and yields are well-known phenomena in the scale-up of processes, due to the increase of heterogeneity of factors such as substrate availability, temperature, pH and oxygen in the bioreactor environment (Bylund et al. 1998; Wang et al. 2020a, b; Du et al. 2022). Furthermore, shear stress on the bacterial cells can occur due to stronger agitation when the bioreactor size increases. The effect of sheer stress on *Lactobacillus delbrueckii* subsp. *bulgaricus* has been observed to be beneficial for cell growth only at intermediate levels and would result in poorer yields at higher shear stress (Arnaud et al. 1993). To circumvent some of these scale-up challenges, the implementation of computational fluid dynamics (CFD) in biological systems can be beneficial (Du et al. 2022). In a 700 L bioreactor cultivating LAB, a soft sensor based on a mechanistic model and CFD were applied as process analytical tools (PAT). Here, the model identified that heterogenous pH zones and loss of biomass within the bioprocess could be reduced by integrating base addition at a centre point of the bioreactor (Spann et al. 2019).

The yield of EPS, however, was higher in the optimised conditions in scale-up. The addition of a live total cell density probe allowed insight into the cell growth, and the bioprocess at 21 °C and 0.18 vvm for optimised EPS production was allowed to continue until the stationary phase at 48 h reaching a final level of 556 mg/L. This longer process time could likely explain some of the higher amounts of EPS, even with a lower biomass yield.

The FTIR analysis of the EPS provided insights into the functional groups present in the isolated polysaccharide. A dominant peak was observed in the region between 800 and 1250 cm⁻^1^ which corresponds to the stretching vibration on C–O–C and C–O which are associated with polysaccharides (Wang and Bi 2008; Tang et al. 2017). This initial structural analysis of the polymer confirms that the EPS is mainly composed of polysaccharides, but further analysis such as NMR, HPLC and SEM could be applied to further elucidate the nature of the structure (Xu et al. 2019; Zhang et al. 2020).

Both *L. rhamnosus* and EPS have been shown in the literature to infer an immunomodulatory response in macrophages (Chabot et al. 2001; Peña and Versalovic 2003). The influence on the secretion of cytokines and chemokines from a murine macrophage when treated with *L. rhamnosus* LRH30, EPS and the whole cell-free fermentation broth was investigated. Our study showed a significant reduction in the secretion of inflammatory cytokines IL-6 and TNF-α, similar to what has been observed in one study when stimulated with strains of *L. rhamnosus* LRH30 (Jorjão et al. 2015). This is in contrast with another study that has observed no effect on IL-6 and TNF-α secretion when stimulated with *L. rhamnosus* cells (Jeffrey et al. 2020). The regulation of chemokines by an *L. rhamnosus* LRH30 from this study is in contrast with a study on pericryptal macrophages that saw an increase in MIP-1β/CCL4 and MIP-2/CXCL2 (Riehl et al. 2019). The immunomodulatory profile of the EPS on macrophages showed an ability to increase IL-10 that was not observed for *L. rhamnosus* LRH30 cells or broth. This effect has also been observed previously (Bleau et al. 2010), and could indicate an ability of the EPS to aid in the treatment of inflammatory conditions (Min 2020). A reduction in IL-6 and TNF-α was observed in the study by Bleau et al (2010). In contrast, our research found that EPS from L. rhamnosus LRH30 reduced IL-6 but had no significant effect on TNF-α.

## Conclusion

This study demonstrates the optimization of the bioprocess parameters temperature and airflow for maximized biomass and EPS production from *L. rhamnosus* LRH30. Experiments were carried out in 250 mL bioreactors, and optimization was obtained through a statistical DOE approach. The optimized conditions identified through a CCD design facilitated high yields of biomass and EPS in both a small-scale 250 mL bioreactor as well as in a 2 L scale-up bioreactor. The structure of the purified EPS produced from *L. rhamnosus* LRH30 produced in a 2 L bioprocess was evaluated through FTIR and showed peaks characteristic of a polysaccharide. The immunomodulatory potential of both products was evidenced by the significantly suppressed secretion of IL-6 and TNF-α from the macrophage when stimulated with LPS and treated with *L. rhamnosus* LRH30 cells. Similarly, EPS was capable of significantly reducing IL-6 secretion, while also stimulating the secretion of IL-10. This highlights the anti-inflammatory potential of both products.

These findings suggest that targeted optimization of the production of *L. rhamnosus* LRH30 and its EPS can yield products with bioactive properties. Further research should be conducted into the scalability of the process to ensure the maintenance of product yield and quality. Finally, this process would be suitable for fed-batch operation, capitalizing on both of the temperature optima by applying the biomass optimised condition in batch and switching to the EPS optimised condition in fed-batch. This approach could enhance the yield of the products, facilitating the way for commercial applications of health-promoting additives.

## Data Availability

No datasets were generated or analysed during the current study.

## References

[CR1] Arnaud JP et al (1993) Shear stress effects on growth and activity of *Lactobacillus delbrueckii* subsp. *bulgaricus*. J Biotechnol 29:157–175 7763707 10.1016/0168-1656(93)90048-r

[CR2] Blainski JML et al (2018) Exopolysaccharides from *Lactobacillus plantarum* induce biochemical and physiological alterations in tomato plant against bacterial spot. Appl Microbiol Biotechnol 102(11):4741–4753. 10.1007/s00253-018-8946-0 29656378 10.1007/s00253-018-8946-0

[CR3] Bleau C et al (2010) Intermediate chains of exopolysaccharides from *Lactobacillus rhamnosus* RW-9595M increase IL-10 production by macrophages. J Appl Microbiol 108(2):666–675. 10.1111/j.1365-2672.2009.04450.x 19702865 10.1111/j.1365-2672.2009.04450.x

[CR4] Bylund F et al (1998) Substrate gradient formation in the large-scale bioreactor lowers cell yield and increases by-product formation. Bioprocess Eng 18(18):171–180

[CR5] Chabot S et al (2001) Exopolysaccharides from *Lactobacillus rhamnosus* RW-9595M stimulate TNF, IL-6 and IL-12 in human and mouse cultured immunocompetent cells, and IFN-γ in mouse splenocytes. Lait 81(6):683–697. 10.1051/lait:2001157

[CR6] Dabour N et al (2006) Improvement of texture and structure of reduced-fat cheddar cheese by exopolysaccharide-producing lactococci. J Dairy Sci 89(1):95–110. 10.3168/jds.S0022-0302(06)72073-2 16357272 10.3168/jds.S0022-0302(06)72073-2

[CR7] Dahiya D, Nigam PS (2023) Dextran of diverse molecular-configurations used as a blood-plasma substitute, drug-delivery vehicle and food additive biosynthesized by *Leuconostoc*, *Lactobacillus* and *Weissella*. Appl Sci 13(22):12526. 10.3390/app132212526

[CR8] Du Y et al (2022) Optimization and scale-up of fermentation processes driven by models. Bioengineering 9(437):1–18 10.3390/bioengineering9090473 PMC9495923 36135019

[CR9] Dubois M et al (1956) Colorimetric method for determination of sugars and related substances. Anal Chem 28(3):350–356. 10.1021/ac60111a017

[CR10] Galle S et al (2010) Exopolysaccharide-forming weissella strains as starter cultures for sorghum and wheat sourdoughs. J Agric Food Chem 58(9):5834–5841. 10.1021/jf1002683 20405917 10.1021/jf1002683

[CR11] Gamar L, Blondeau K, Simonet JM (1997) Physiological approach to extracellular polysaccharide production by *Lactobacillus rhamnosus* strain C83. J Appl Microbiol 83(3):281–287. 10.1046/j.1365-2672.1997.00228.x

[CR12] Gamar-Nourani L, Blondeau K, Simonet JM (1998) Influence of culture conditions on exopolysaccharide production by *Lactobacillus rhamnosus* strain C83. J Appl Microbiol 85(4):664–672. 10.1111/j.1365-2672.1998.00574.x

[CR13] Gorbach SL (1990) Lactic acid bacteria and human health. Ann Med 22(1):37–41. 10.3109/07853899009147239 2109988 10.3109/07853899009147239

[CR14] Gorbach SL et al. (1989) *Lactobacillus* strains and methods of selection. US patent 4839281A. Systems and methods for robotic gutter cleaning along an axis of rotation

[CR15] Jeffrey MP et al (2020) Milk fermented with Lactobacillus rhamnosus R0011 induces a regulatory cytokine profile in LPS-challenged U937 and THP-1 macrophages. Curr Res Food Sci 3:51–58. 10.1016/j.crfs.2020.02.002 32914120 10.1016/j.crfs.2020.02.002 PMC7473351

[CR16] Jorjão AL et al (2015) Live and heat-killed *Lactobacillus rhamnosus* ATCC 7469 may induce modulatory cytokines profiles on macrophages RAW 264.7. Sci World J. 10.1155/2015/716749 10.1155/2015/716749 PMC4663741 26649329

[CR17] Liang Z et al (2010) Extraction and structural characteristics of extracellular polymeric substances (EPS), pellets in autotrophic nitrifying biofilm and activated sludge. Chemosphere 81(5):626–632. 10.1016/j.chemosphere.2010.03.043 20655088 10.1016/j.chemosphere.2010.03.043

[CR18] London LEE et al (2015) Use of *Lactobacillus mucosae* DPC 6426, an exopolysaccharide-producing strain, positively influences the techno-functional properties of yoghurt. Int Dairy J 40:33–38. 10.1016/j.idairyj.2014.08.011

[CR19] Macedo MG et al (2002) Effect of medium supplementation on exopolysaccharide production by *Lactobacillus rhamnosus* RW-9595M in whey permeate. Int Dairy J 12(5):419–426. 10.1016/S0958-6946(01)00173-X 10.1021/bp0101637 11934282

[CR20] Medve B, Lipt D (2008) Characterization of the growth of *Lactobacillus rhamnosus* GG in milk at suboptimal temperatures. J Food Nutr Res 47(2):60–67

[CR21] Min Z et al (2020) Exopolysaccharides from *Lactobacillus plantarum* YW11 improve immune response and ameliorate inflammatory bowel disease symptoms. Acta Biochim Pol 67(4):487–493. 10.18388/ABP.2020_5171 10.18388/abp.2020_5371 33332076

[CR22] Moscovici M (2015) Present and future medical applications of microbial exopolysaccharides. Front Microbiol 6(SEP):1–11. 10.3389/fmicb.2015.01012 26483763 10.3389/fmicb.2015.01012 PMC4586455

[CR23] Myers RH (1999) Response surface methodology—current status and future directions response surface methodology—current status and future directions. J Qual Technol 31(1):30–41. 10.1080/00224065.1999.11979891

[CR24] Nguyen PT et al (2020) Exopolysaccharide production by lactic acid bacteria: the manipulation of environmental stresses for industrial applications. AIMS Microbiol 6(4):451–469. 10.3934/MICROBIOL.2020027 33364538 10.3934/microbiol.2020027 PMC7755584

[CR25] Oleksy-Sobczak M, Klewicka E, Piekarska-Radzik L (2020) Exopolysaccharides production by *Lactobacillus rhamnosus* strains—optimization of synthesis and extraction conditions. Lwt. 10.1016/j.lwt.2020.109055 10.1007/s12602-019-09581-2 PMC7306023 31410767

[CR26] Peña JA, Versalovic J (2003) *Lactobacillus rhamnosus* GG decreases TNF-α production in lipopolysaccharide-activated murine macrophages by a contact-independent mechanism. Cell Microbiol 5(4):277–285. 10.1046/j.1462-5822.2003.t01-1-00275.x 12675685 10.1046/j.1462-5822.2003.t01-1-00275.x

[CR27] Perry DB, Mcmahon DJ, Oberg CJ (1997) Effect of exopolysaccharide-producing cultures on moisture retention in low fat mozzarella cheese. J Dairy Sci 80(5):799–805. 10.3168/jds.S0022-0302(97)76000-4

[CR28] Puebla-Barragan S, Reid G (2019) Forty-five-year evolution of probiotic therapy. Microbial Cell 6(4):184–196. 10.15698/mic2019.04.673 30956971 10.15698/mic2019.04.673 PMC6444557

[CR29] Rehman ZU, Vrouwenvelder JS, Saikaly PE (2021) Physicochemical properties of extracellular polymeric substances produced by three bacterial isolates from biofouled reverse osmosis membranes. Front Microbiol 12(July):1–13. 10.3389/fmicb.2021.668761 10.3389/fmicb.2021.668761 PMC8328090 34349735

[CR30] Riehl TE et al (2019) Lactobacillus rhamnosus GG protects the intestinal epithelium from radiation injury through release of lipoteichoic acid, macrophage activation and the migration of mesenchymal stem cells. Gut Microbiota 68(6):1003–1013. 10.1136/gutjnl-2018-316226 10.1136/gutjnl-2018-316226 PMC7202371 29934438

[CR31] Roberfroid MB (2002) Global view on functional foods: European perspectives. Br J Nutr 88:133–138. 10.1079/BJN2002677 12495454 10.1079/BJN2002677

[CR32] Ryan PM et al (2015) Sugar-coated: exopolysaccharide producing lactic acid bacteria for food and human health applications. Food Funct 6(3):679–693. 10.1039/c4fo00529e 25580594 10.1039/c4fo00529e

[CR33] Saleem M et al (2021) Isolation and functional characterization of exopolysaccharide produced by *Lactobacillus plantarum* S123 isolated from traditional Chinese cheese. Arch Microbiol 203(6):3061–3070. 10.1007/s00203-021-02291-w 33791833 10.1007/s00203-021-02291-w

[CR34] Sørensen HM et al (2022) ‘Exopolysaccharides of lactic acid bacteria: production, purification and health benefits towards functional food. Nutrients. 10.3390/nu14142938 35889895 10.3390/nu14142938 PMC9319976

[CR35] Sørensen HM, Cunningham D et al (2023) ‘Steps toward a digital twin for functional food production with increased health benefits. Curr Res Food Sci. 10.1016/j.crfs.2023.100593 37790857 10.1016/j.crfs.2023.100593 PMC10543970

[CR36] Sørensen HM, Rochfort KD et al (2023) Bioactive ingredients from dairy-based lactic acid bacterial fermentations for functional food production and their health effects. Nutrients 15(22):4754. 10.3390/nu15224754 38004148 10.3390/nu15224754 PMC10675170

[CR37] Sørensen HM et al (2024) Optimisation of dairy-based media using response surface modelling to increase yields of *Lacticaseibacillus rhamnosus* promoting an enhanced immune response. Int Dairy J 156:105990. 10.1016/j.idairyj.2024.105990

[CR38] Spann R, Gernaey KV, Sin G (2019) A compartment model for risk-based monitoring of lactic acid bacteria cultivations. Biochem Eng J 151(January):107293. 10.1016/j.bej.2019.107293

[CR39] Tamang JP et al (2020) Fermented foods in a global age: East meets West. Compr Rev Food Sci Food Saf 19(1):184–217. 10.1111/1541-4337.12520 33319517 10.1111/1541-4337.12520

[CR40] Tang J et al (2017) Distinguishing the roles of different extracellular polymeric substance fractions of a periphytic biofilm in defending against Fe_2_O_3_ nanoparticle toxicity. Environ Sci Nano 4(8):1682–1691. 10.1039/c7en00352h

[CR41] Wang M, Bi J (2008) Modification of characteristics of kefiran by changing the carbon source of *Lactobacillus kefiranofacien*. J Sci Food and Agric 88(2):125–135. 10.1002/jsfa

[CR42] Wang Y et al (2008) Physicochemical properties of exopolysaccharide produced by *Lactobacillus kefiranofaciens* ZW3 isolated from Tibet kefir. Int J Biol Macromol 43(3):283–288. 10.1016/j.ijbiomac.2008.06.011 18662712 10.1016/j.ijbiomac.2008.06.011

[CR43] Wang G et al (2020a) Developing a computational framework to advance bioprocess scale-up. Trends Biotechnol 38(8):846–856. 10.1016/j.tibtech.2020.01.009 32493657 10.1016/j.tibtech.2020.01.009

[CR44] Wang T et al (2020b) Fermentation optimization and kinetic model for high cell density culture of a probiotic microorganism: *Lactobacillus rhamnosus* LS - 8. Bioprocess Biosyst Eng 43(3):515–528. 10.1007/s00449-019-02246-y 31712884 10.1007/s00449-019-02246-y

[CR45] Xu Y et al (2019) Purification, characterization and bioactivity of exopolysaccharides produced by *Lactobacillus plantarum* KX041. Int J Biol Macromol 128:480–492. 10.1016/j.ijbiomac.2019.01.117 30682478 10.1016/j.ijbiomac.2019.01.117

[CR46] Zhang W-p et al (2020) Structural characterization and induced copper stress resistance in rice of exopolysaccharides from *Lactobacillus plantarum* LPC-1. Int J Biol Macromol 152:1077–1088. 10.1016/j.ijbiomac.2019.10.195 31751733 10.1016/j.ijbiomac.2019.10.195

